# *Cosmc* deficiency causes spontaneous autoimmunity by breaking B cell tolerance

**DOI:** 10.1126/sciadv.abg9118

**Published:** 2021-10-06

**Authors:** Junwei Zeng, Rajindra P. Aryal, Kathrin Stavenhagen, Chi Luo, Renyan Liu, Xiaohui Wang, Jiaxuan Chen, Hao Li, Yasuyuki Matsumoto, Yingchun Wang, Jianmei Wang, Tongzhong Ju, Richard D. Cummings

**Affiliations:** 1 Department of Surgery, Beth Israel Deaconess Medical Center, Harvard Medical School, Boston, MA, USA.; 2 Department of Cancer Biology, Dana-Farber Cancer Institute, Boston, MA, USA.; 3 Department of Medical Oncology, Dana-Farber Cancer Institute, Boston, MA, USA.; 4 Department of Developmental Biology, Harvard School of Dental Medicine, Boston, MA, USA.; 5 Department of Medicine, Beth Israel Deaconess Medical Center, Harvard Medical School, Boston, MA, USA.; 6 Department of Biochemistry, Emory University, Atlanta, GA, USA.

## Abstract

Factors regulating the induction and development of B cell–mediated autoimmunity are not well understood. Here, we report that targeted deletion in murine B cells of X-linked *Cosmc*, encoding the chaperone required for expression of core 1 O-glycans, causes the spontaneous development of autoimmune pathologies due to a breakdown of B cell tolerance. BC-*Cosmc*KO mice display multiple phenotypic abnormalities, including severe weight loss, ocular manifestations, lymphadenopathy, and increased female-associated mortality. Disruption of B cell tolerance in BC-*Cosmc*KO mice is manifested as elevated self-reactive IgM and IgG autoantibodies. *Cosmc*-deficient B cells exhibit enhanced basal activation and responsiveness to stimuli. There is also an elevated frequency of spontaneous germinal center B cells in BC-*Cosmc*KO mice. Mechanistically, loss of *Cosmc* confers enhanced B cell receptor (BCR) signaling through diminished BCR internalization. The results demonstrate that *Cosmc*, through control of core 1 O-glycans, is a previously unidentified immune checkpoint gene in maintaining B cell tolerance.

## Teaser

O-glycans on B cell glycoproteins controlled by X-linked *Cosmc* are required for B cell tolerance and preventing autoimmunity.

## INTRODUCTION

B cells play pivotal roles in mediating the pathogenesis of autoimmune diseases (AIDs), which affect ~7 to 9% of the world population (*1*–*3*). A hallmark of autoimmunity is the breach of B cell tolerance, where B cells are unable to distinguish self-antigens from non–self-antigens and eventually lead to the emergence of pathogenic autoantibodies. Nonetheless, the molecular factors underneath the initial development and expansion of pathogenic autoreactive B cells remain incompletely understood.

The B cell receptor (BCR) is the master regulator in controlling B cell development, differentiation, survival, and tolerance (*1*, *4*, *5*). It is essential for B cells to receive, process, and integrate both the antigenic signals delivered via BCR and environmental cues relayed via other receptors as they give rise to opposing outcomes. The B cells are either appropriately activated and generate high-affinity antibodies against foreign antigens, or they become tolerogenic upon interaction with self-antigens. Multiple physiological processes to keep autoreactive B cells in check have been found (*6*–*9*). Nonetheless, in the periphery, a notable percentage of B cells in healthy host exhibit autoreactivities (*7*, *10*), suggesting the existence of undefined mechanisms underlying the fine-tuned regulation of B cell activities.

Glycosylation is an important posttranslational modification (PTM) that has been implicated in multiple AIDs (*11*–*17*). Abnormally glycosylated immunoglobulins have been found in immunoglobulin A (IgA) nephropathy (IgAN), systemic lupus erythematosus, and other AIDs. Interactions between sialic acid–binding immunoglobulin type lectins (Siglecs) and sialic acids are involved in autoimmunity by regulating BCR and Toll-like receptor (TLR) signaling. In response to both developmental and environmental cues, dynamic glycosylation has been well documented on immune cells and molecules. In the context of AID pathogenesis, such dynamic glycosylation changes might play roles in mediating the initiation and progression of AID but are largely unexplored.

A major PTM of surface proteins in B cells is the addition of O-glycans through formation of core 1 O-glycans (R-Galβ1-3GalNAcα1-Ser/Thr/Tyr). The generation of O-glycans, which are abundantly present on leukocytes (*18*–*22*), requires expression of both *Cosmc* (*C1GalT1C1*), a molecular chaperone in the endoplasmic reticulum, and its only client, the Core 1 β3galactosyl-tranferase (*C1GalT1*), also known as T-synthase. We recently demonstrated that deletion of *Cosmc* in murine B cells, which results in expression of the Tn antigen (GalNAcα1-Ser/Thr/Tyr), greatly reduces their migration to lymph nodes (*23*). We also observed that the B cell-specific *Cosmc*-knockout (BC-*Cosmc*KO) mice displayed splenomegaly and hypergammaglobulinemia, indicating the potential development of AID. *Cosmc* is an especially interesting candidate gene, as it has been recently implicated in several autoimmune and inflammation-associated diseases, including inflammatory bowel diseases (*24*), IgAN (*25*), Tn syndrome (*26*), and Alzheimer’s disease (*27*).

Here, we report that *Cosmc* deficiency in B cells causes a breakdown of B cell tolerance. BC-*Cosmc*KO mice spontaneously display multiple AID-like pathological phenotypes, in an age- and gender-associated manner. *Cosmc* deficiency prolongs the retention of the BCR on cell surface and promotes stronger BCR signaling, which likely led to B cells’ hyperresponsiveness to stimuli. These results demonstrate that the presence of *Cosmc*, required for normal O-glycans on B cell glycoproteins, controls B cell tolerance by maintaining BCR signaling through regulating surface BCR internalization.

## RESULTS

### AID-like pathological features in BC-*Cosmc*KO mice

**Fig. 1.**
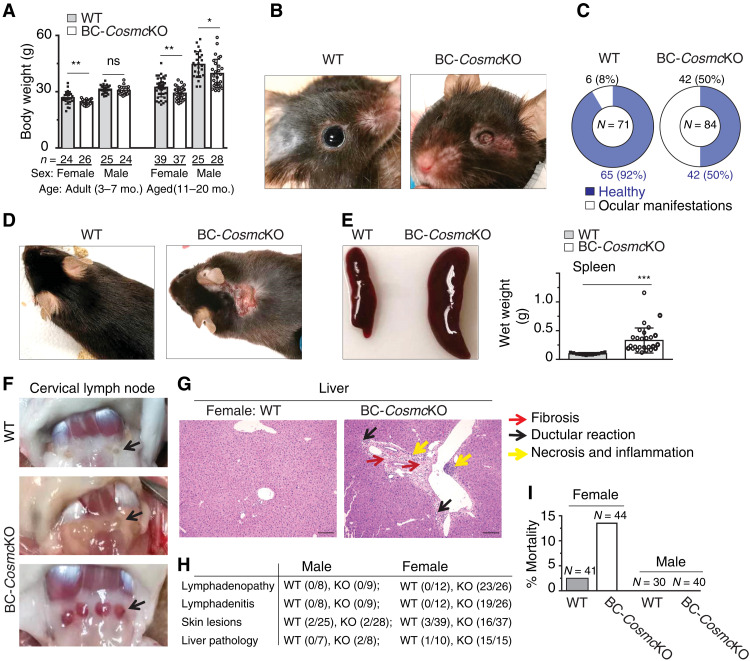
AID-like pathological features in BC-*Cosmc*KO mice. Obvious phenotypic abnormalities of aged [11 to 20 months (mo.)] female and male mice from both BC-*Cosmc*KO and WT littermate controls were documented (**A** to **F**). Body weight of both adult (3 to 7 months) and aged mice from both genders is shown (A): adult female: WT 26.75 ± 3.37, BC-*Cosmc*KO 24.58 ± 1.26; adult male: WT 31.25 ± 2.15, BC-*Cosmc*KO 30.57 ± 2.09; aged female: WT 32.26 ± 5.06, BC-*Cosmc*KO 29.14 ± 3.27; aged male: WT 44.48 ± 6.92, BC-*Cosmc*KO 39.61 ± 7.68. Each symbol (black square, WT; open circle, BC-*Cosmc*KO) represents an individual mouse body weight, graphed as means ± 1 SD. Ocular manifestations (B and C) and dermatitis (D). Spleens (E) and cervical lymph nodes (F) of aged female mice. Representative images from both WT and BC-*Cosmc*KO mice are shown in (B) to (F); *n* = 12 for WT and *n* = 26 for BC-*Cosmc*KO (E). (**G**) Liver damage in aged female *Cosmc* mutant mice. Liver sections were stained with hematoxylin and eosin (H&E). Yellow arrows indicate necrosis and inflammation. Black arrows indicate ductular reaction. Red arrows indicate fibrosis. (**H**) Summary of examined animals in (D), (F), and (G). Liver H&E images were acquired at ×20 magnification. Scale bars, 100 μm. (**I**) Bar graphs represent mortality summary of both BC-*Cosmc*KO and WT littermate controls. Each symbol (black square, WT; open circle, BC-*Cosmc*KO) represents an individual mouse, graphed as means ± 1 SEM. ns, not significant. Unpaired two-tailed Student’s *t* tests were performed to determine statistical significance, *P < 0.05, **P < 0.01, and ***P < 0.001. Photo credit: Junwei Zeng, Beth Israel Deaconess Medical Center.

A range of obvious phenotypic abnormalities (Fig. 1) spontaneously developed in aged (11 to 20 months) BC-*Cosmc*KO mice, although they appeared healthy at younger ages (8 weeks). Significant weight loss occurred in adult (3 to 7 months) female and both gender groups in aged BC-*Cosmc*KO mice (Fig. 1A); however, the severity of the weight loss was greater in female mutant mice. Moreover, a substantial portion of mutant mice displayed ocular abnormalities in a syndromic fashion, ranging in severity from cataracts, keratitis, and watery eyes to a complete eye closure or loss of the eyeball in some mutant mice (Fig. 1, B and C, and fig. S1, A to C). In addition, a higher incidence of dermatitis occurred in aged female BC-*Cosmc*KO mice (Fig. 1, D and H).

We observed enlarged spleen in young (2 to 4 months) (*23*) but not in aged (11 to 20 months) male BC-*Cosmc*KO mice (fig. S1D, left). By contrast, female mice had significantly larger spleens in both young (2 to 4 months) and older aged groups (11 to 20 months) (Fig. 1E and fig. S1D, right). Aged BC-*Cosmc*KO female mice also exhibited lymphadenopathy and lymphadenitis (Fig. 1, F and H). Notably, ~73% of the lymph nodes exhibited severe inflammation (Fig. 1F, bottom). Tissue pathological features were evident in the liver of aged female mutant mice, characterized by fibrosis, ductular reaction, and necrosis and inflammation (Fig. 1, G, right, and H). However, the liver of aged male mutant mice was mildly affected, and the animals exhibited resistance to age-associated hepatic steatosis (fig. S1E).

By 18 months, 14% (6 of 44) of female mutant mice became moribund, whereas only 2% (1 of 41) of wild type (WT) expired (Fig. 1I). Together, BC-*Cosmc*KO mice exhibited various tissue pathologies that are typically associated with AIDs, with a strong female bias.

### Loss of B cell tolerance in BC-*Cosmc*KO mice

**Fig. 2.**
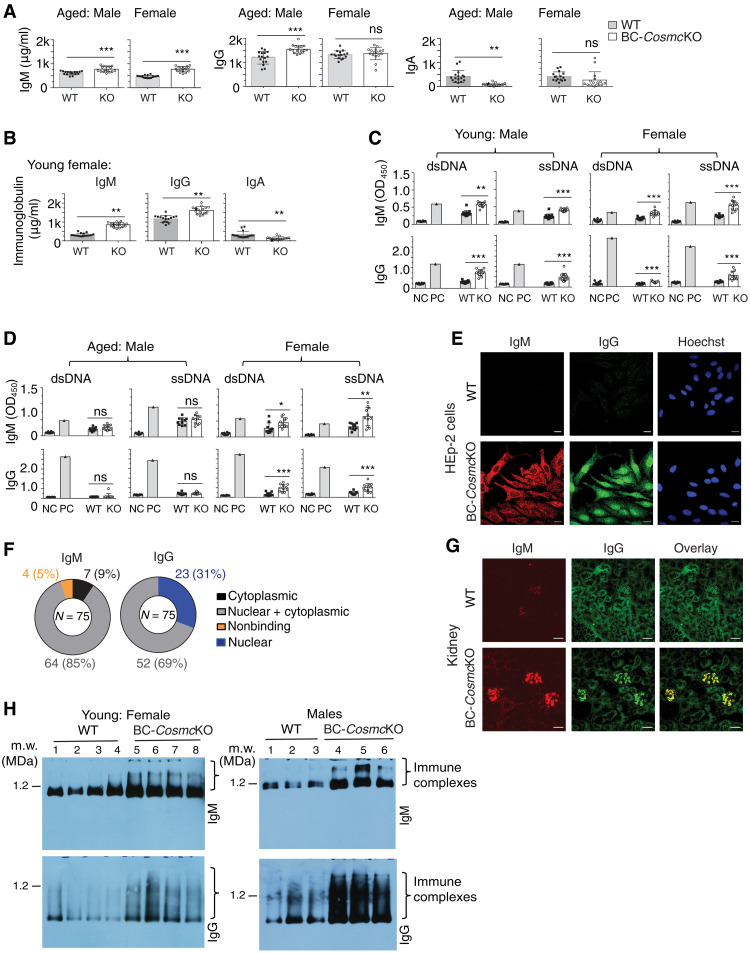
Loss of *Cosmc* breaks B cell tolerance. Total immunoglobulins (**A** and **B**) and anti-dsDNA and anti-ssDNA antibodies (**C** and **D**) in sera from both BC-*Cosmc*KO and WT littermate controls measured by ELISA. NC, negative control (media only); PC, positive control (MRL/lpr serum). All groups include 16 WT and 16 BC-*Cosmc*KO mice in (A) and (B) and 10 WT and 11 BC-*Cosmc*KO mice in (C) and (D), except for 14 WT and 15 BC-*Cosmc*KO mice in the young male group. KO, BC-*Cosmc*KO. Each symbol (black square, WT; open circle, BC-*Cosmc*KO) represents an individual mouse, graphed as means ± 1 SEM. Unpaired two-tailed Student’s *t* tests were performed to determine statistical significance, *P < 0.05, **P < 0.01, and ***P < 0.001. (**E**) Autoreactive antibodies of BC-*Cosmc*KO mice. Serum samples (1:90 dilution) from BC-*Cosmc*KO and WT littermate controls assayed by HEp-2 cell staining. Images were acquired at ×63 magnification. Representative images from young male WT and BC-*Cosmc*KO mice are shown in (E). Scale bars, 20 μm. (**F**) Summary of assayed samples from 2- to 4-month-old BC-*Cosmc*KO mice of both genders. (**G**) Immunoglobulin deposition in kidneys of male BC-*Cosmc*KO mice. Kidney images were acquired at ×20 magnification. Representative images from both WT (*n* = 4) and BC-*Cosmc*KO male mice (*n* = 4) are shown. Scale bars, 50 μm. (**H**) BN-APAGE analysis of mouse serum. IgM (top) and IgG (bottom) of four young female and three male mice of each genotype are shown. Serum or tissues were collected from aged (11 to 20 months old) and young (2 to 4 months old) mice of indicated gender.

We observed an increased level of total IgM and IgG and a reduced level of IgA in the sera of young (2 months) and aged (11 to 20 months) male BC-*Cosmc*KO mice (Fig. 2A) (*23*), and an altered immunoglobulin profile was also observed in young female BC-*Cosmc*KO mice (Fig. 2B). By contrast, compared to WT, aged (11 to 20 months) female BC-*Cosmc*KO mice exhibited increased serum IgM levels. However, total IgG or IgA levels were unaltered (Fig. 2A). To examine whether the B cell tolerance was impaired in the mutant mice, we measured the titers of immunoglobulins that recognize double-stranded and single-stranded DNA (dsDNA and ssDNA). We observed increased titers of anti-dsDNA and anti-ssDNA IgG and IgM in female BC-*Cosmc*KO mice of both young (Fig. 2C, right) and aged groups (Fig. 2D, right). Such increased autoreactive antibody levels were only observed in young male (Fig. 2C, left) but not in old male BC-*Cosmc*KO mice (Fig. 2D, left). These results demonstrate that BC-*Cosmc*KO mice produce autoreactive antibodies, indicating a breach in B cell self-tolerance.

As a more comprehensive assessment of self-reactivity, we collected sera from both WT and BC-*Cosmc*KO mice (2 months) to test the autoantigen specificity by an indirect immunofluorescence assay using HEp-2 cells, a method commonly used to identify autoreactive antibodies. While sera from WT mice had minimal reactivity, sera from all BC-*Cosmc*KO mice (75 of 75) demonstrated autoreactivities and yielded multiple staining patterns (Fig. 2E and fig. S2A, right). Moreover, consistent with the enhanced autoreactive IgM titers in mutant mice shown by enzyme-linked immunosorbent assay (ELISA), the staining specificity was not only restricted to IgG antibodies but was also observed with IgM, suggesting an early break of B cell tolerance in *Cosmc*-deficient B cells (Fig. 2E and fig. S2A). We observed that sera from 69% of the BC-*Cosmc*KO animals exhibited self-reactive IgG with a nuclear/cytoplasmic staining pattern, whereas the remaining 31% exclusively stained nuclei. By contrast, for IgM, 85% of the BC-*Cosmc*KO mice sera displayed IgM nuclear/cytoplasmic staining, 9% stained the cytoplasm, and 5% displayed no staining (Fig. 2F). There was a higher percentage of autoreactive IgG staining nuclei in sera from female BC-*Cosmc*KO mice (47% versus 25% in male) (fig. S2B). Notably, staining by IgM did not necessarily reflect the same staining pattern as the IgG of the same BC-*Cosmc*KO mouse. The wide spectrum of self-reactive staining patterns of immunoglobulins from BC-*Cosmc*KO mice indicates that they have a breakdown of B cell tolerance. Immunofluorescence staining analysis on kidney sections of the mutant mice also showed notable deposits of IgG and IgM antibodies when compared to WT (Fig. 2G and fig. S2C), suggesting potential immune complex deposition in tissues. Nonetheless, aged BC-*Cosmc*KO mice showed similar range of urinary protein and other substances to those in the WT (fig. S2, D and E), indicating normal renal function. In addition, histopathological evaluation shows the absence of severe kidney structural changes in aged males, with slightly higher glomerular alteration scores in aged female BC-*Cosmc*KO mice (fig. S2, F and G).

The uncontrolled autoantibodies may contribute to disease progression by multiple effector mechanisms (*28*, *29*). The high titers of autoantibodies in sera of BC-*Cosmc*KO mice prompted us to examine the presence of circulating immune complexes, as immune complexes are formed during the progressive course of many human diseases, including AIDs (*30*, *31*). We used the blue native–agarose polyacrylamide gel electrophoresis (BN-APAGE) system, which resolves native protein complexes up to 6 MDa or greater (*32*). A range of immune complexes of IgG and IgM, with varying molecular weights, was observed in the sera of the young (2 months) mutant female and male mice (Fig. 2H). However, aged mice (11 to 20 months) did not show consistent differences in the presence of such macromolecular immune complexes (fig. S2H). It is conceivable that over time, immune complexes may progressively precipitate in tissues or lessen in blood. Inflammatory cytokines have been implicated in the development of AIDs (*33*, *34*). In the present study, we observed a mild but significant increase of tumor necrosis factor–α (TNFα) but not interferon-γ (IFNγ) in the sera of aged female *Cosmc* mutant mice, indicating a state of low-grade, chronic inflammation in these mice (fig. S3, A and B). The complement system is another downstream effector arm of autoantibody-mediated injury (*35*, *36*). Complement C3 is the convergent point of the classic, lectin, and alternative pathways and has been implicated in autoimmunity. Western blot analyses showed an increased circulating level of C3 in the sera of several aged female BC-*Cosmc*KO mice, concomitantly with reduced C3 breakdown products (fig. S3, C and D). The irregular presence of C3 and its split products indicates that the complement cascade might play a role in the pathogenesis of the observed disease in aged female BC-*Cosmc*KO mice. Together, these results indicate that deletion of *Cosmc* in B cells disrupts B cell tolerance, which is associated with altered immune effector factors.

### Hyperresponsiveness of *Cosmc*-deficient B cells

**Fig. 3.**
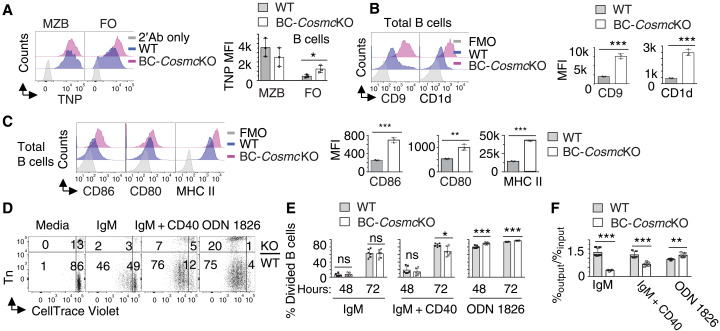
Hyperresponsiveness of *Cosmc*-deficient B cells. (**A**) Representative histogram plots of TNP-Ficoll antigen binding on WT and Cosmc-deficient B cells. Right: Mean fluorescence intensity (MFI) of anti-TNP antibody on B cells shown for each WT and BC-*Cosmc*KO mouse. Enhanced expression of CD9 and CD1d (**B**) and CD86, CD80, and MHC II (**C**) on *Cosmc*-deficient B cells. Right: MFI of indicated molecules on B cells is shown. FMO, fluorescence minus one control. For (A) to (C), results are representative of three independent experiments with at least three animals in each group. Splenocytes were prepared from both BC-*Cosmc*KO and WT littermate controls. (**D**) Representative fluorescence-activated cell sorting (FACS) analysis of splenocyte coculture system at day 3. FACS plots are from a gated CD19^+^ population. Mixed splenocytes from both BC-*Cosmc*KO and WT littermate controls were labeled with CellTrace Violet and cultured in the same well in the presence of indicated stimuli. BC-*Cosmc*KO B cells were stained positive with anti-Tn antibody. CellTrace dilution indicates cell division. Quantification of divided cells of BC-*Cosmc*KO (Tn^+^) or WT (Tn^−^) B cells is shown (**E**). Cell viability measured by the ratio of [percentage of dye^+^ Tn^−^ or Tn^+^ B cells]_output_/[total B cells]_output_ to that of input and quantified (**F**). Results are from three independent experiments. Each symbol (black square, WT; open circle, BC-*Cosmc*KO) represents an individual mouse, graphed as means ± 1 SEM. Young (2 to 4 months old) male mice were used in (A) to (F). Unpaired two-tailed Student’s *t* tests were performed to determine statistical significance, *P < 0.05, **P < 0.01, and ***P < 0.001.

Disrupted B cell tolerance is often associated with irregular B cell development and B cell hyperresponsiveness (*2*, *37*). We have observed altered percentage and numbers of marginal zone (MZB) and follicular (FO) B cell in the spleen of BC-*Cosmc*KO mice (*23*). To functionally characterize these B cell subsets, we used flow cytometry to measure their uptake of a model antigen trinitrophenol (TNP)–Ficoll, which is a T-independent antigen that is quickly trapped by MZB and marginal zone precursor (MZP) after intravenous injection (*38*). We observed comparable binding of TNP-Ficoll in both WT and BC-*Cosmc*KO MZB cells, functionally confirming that the increased B cell subset in BC-*Cosmc*KO mice is MZB cells (*23*). By contrast, *Cosmc*-deficient FO B cells demonstrated significantly higher ability to bind to TNP-Ficoll (Fig. 3A). Furthermore, total *Cosmc*-deficient B cells (Fig. 3B) and subsets (fig. S4A) also showed higher expression of CD9 and CD1d, markers for MZB cells. Together, these data indicated that loss of *Cosmc* affects the differentiation of splenic B cells toward the MZB cell pathway.

We have observed enhanced basal immunoglobulin amounts in BC-*Cosmc*KO mice (Fig. 2A and fig. S2A) (*23*), which may result from a generalized hyperactivation of B cells. Flow cytometry analysis indicated that *Cosmc*-deficient B cells display enhanced basal activation, as measured by the surface expression of activation markers, including CD86, CD80, and major histocompatibility complex class II (MHC II) (Fig. 3C and fig. S4, B and C). Furthermore, we explored whether *Cosmc* mutation affects B cell responsiveness to anti-IgM in the absence or presence of anti-CD40 and oligodeoxynucleotide 1826 (ODN 1826) stimulation in vitro. By measuring CellTrace Violet dilution, we observed that *Cosmc*-deficient B cells behaved similarly to WT B cells in terms of cell division upon anti-IgM stimulation. The addition of anti-CD40 stimulation, however, led to reduced division in *Cosmc*-deficient B cells (Fig. 3, D and E), compared to WT. *Cosmc*-deficient B cells showed impaired viability compared to WT cells upon anti-IgM activation, indicating elevated sensitivity to BCR cross-linking; the difference in viability was reduced in the presence of anti-CD40 (Fig. 3, D and F). By contrast, ODN 1826 alone induced significantly more division and expansion of *Cosmc*-deficient B cell (Fig. 3, D to F). These results indicate that *Cosmc*-deficient B cells are hyperresponsive to BCR stimulation (in vivo and in vitro), as well as other signals, such as a TLR signal. This altered responsiveness, together with the perturbed B cell differentiation, is reminiscent of those found in autoreactive B cells seen in AID patient and animal models (*37*, *39*, *40*).

### *Cosmc* deficiency leads to increased spontaneous germinal center B cells

**Fig. 4.**
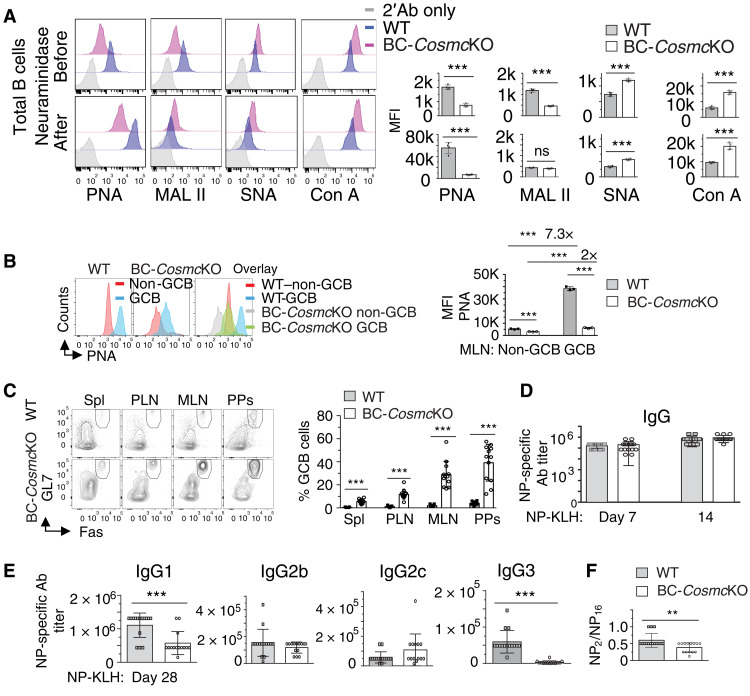
Enhanced spontaneous GCB cells in BC-*Cosmc*KO mice. Binding analysis of biotinylated lectins PNA, MAL II, SNA, and Con A, followed by streptavidin–Alexa Fluor 488, on neuraminidase- or PBS-treated WT and BC-*Cosmc*KO splenic B cells (**A**). PNA binding of GCB cells (B220^+^ or CD19^+^ GL7^+^ Fas^+^) and non-GCB cells (B220^+^ or CD19^+^ GL7^−^ Fas^−^) (**B**). Right: MFI of lectin binding on indicated B cell population is shown for each WT and BC-*Cosmc*KO mouse in (A) and (B). (**C**) Representative FACS plots of GCB cells in BC-*Cosmc*KO mice in indicated tissues. Right: Percentage of GCB cells of total B cells is shown for each WT and BC-*Cosmc*KO mouse. (**D** and **E**) NP-specific immunoglobulin responses to immunization with NP-KLH. (**F**) Ratio of NP-specific high-affinity IgG to total NP-specific IgG was from two experiments with similar results. In (D), *n* = 15 for both WT and BC-*Cosmc*KO mice. In (E) and (F), *n* = 15 for both WT and *n* = 14 for BC-*Cosmc*KO mice at day 28. Representative plots from both WT and BC-*Cosmc*KO mice are shown in (A) to (C). Each symbol (black square, WT; open circle, BC-*Cosmc*KO) represents an individual mouse, graphed as means ± 1 SEM. Young (2 to 4 months old) male mice were used in (A) to (F). Unpaired two-tailed Student’s *t* tests were performed to determine statistical significance, **P < 0.01 and ***P < 0.001.

Germinal center B (GCB) cells play essential roles in maintaining B cell tolerance (*41*, *42*). The first surface marker historically to identify GCB cells was high binding of peanut agglutinin (PNA) (*43*, *44*), a plant lectin that specifically binds to the core 1 O-glycan, Galβ1-3GalNAcα1-Ser/Thr-R. The rapid increase of PNA binding on GCB cells suggests that once B cells differentiate and take on GCB cell features, abundant core 1 O-glycans are concomitantly expressed on the cell surface. Nonetheless, the physiological relevance of this sudden increase of core 1 O-glycans on GCB cells remains completely unknown. Notably, the galactose in core 1 O-glycans is added by T-synthase, an enzyme requiring *Cosmc* for its folding and consequent activity (*20*–*22*). To gain insights into the significance of PNA ligands on GCB cells, we characterized glycans on naïve splenic WT and *Cosmc*-deficient B cells by using a panel of lectins that selectively recognize different glycan structures (Fig. 4A and fig. S5A). As expected, WT B cells bound PNA substantially higher than *Cosmc*-deficient B cells with or without neuraminidase treatment, by which removal of sialic acids can expose the core 1 O-glycans. Consistent with this, we observed that naïve WT B cells are strongly bound by MAL II, a lectin which recognizes the sialylated core 1 O-glycan; binding was decreased after neuraminidase treatment. By comparison, *Cosmc*-deficient B cells exhibited low MAL II binding under both conditions. We observed that *Cosmc*-deficient B cells display higher binding of the lectin Con A, which recognizes α-mannosyl structures on N-glycans, and higher binding of *Sambucus nigra* agglutinin (SNA), which binds to Siaα2,6 structure on N-glycans; such glycans might be more accessible on cell surfaces as a result of loss of extended O-glycans. These results indicate that the glycans recognized by PNA on B cells are core 1 O-glycans, whose expression is decreased on *Cosmc*-deficient B cells. When GCB cells (B220^+^Fas^+^GL7^+^) from mesenteric lymph node (Fig. 4B) or spleen (fig. S5B) were stained with PNA and binding assessed by mean fluorescence intensity (MFI), there was substantially higher PNA binding to WT GCB cells compared to *Cosmc*-deficient GCB cells. We found a substantially increased percentage of spontaneous GCB cells in all lymphoid tissues of BC-*Cosmc*KO mice (Fig. 4C). Similar results with a higher percentage of GCB cell population were also found in BC-*Cosmc*KO mice, using another set of GCB cell marker CD38^lo^ and Fas^+^ (fig. S5C). These data indicate that *Cosmc* plays a negative regulatory role in the development of GCB cells.

*Cosmc*-deficient B cells retained their ability to generate antibodies specific to type I and type II T-independent immunogens 4-hydroxy-3-nitrophenylacetyl–conjugated lipopolysaccharide (NP-LPS) and NP-Ficoll, respectively, and T-dependent immunogen NP-conjugated keyhole limpet hemocyanin (NP-KLH). Significant difference in NP-specific IgM level at an early stage was observed when animals were immunized with NP-Ficoll, a chemical agent that activates MZB cells (fig. S6, A and B). By contrast, after challenge with NP-KLH, comparable NP-specific IgG levels were observed in BC-*Cosmc*KO mice at early stages (Fig. 4D). Later on, at day 28, the mutant mice expressed a reduced NP-specific IgG1 titer and a negligible IgG3 response to NP-KLH (Fig. 4E). These results mirror our previously observed difference in basal serum IgG3 level, which was extremely low in BC-*Cosmc*KO mice (*23*). These data indicate that *Cosmc* plays an essential role in mediating isotype switching to IgG3. Furthermore, we observed a notable reduction in the level of high-affinity NP-specific IgG in BC-*Cosmc*KO mice at day 28 after NP-KLH immunization (Fig. 4F).

### Impaired BCR internalization of *Cosmc*-deficient B cells

**Fig. 5.**
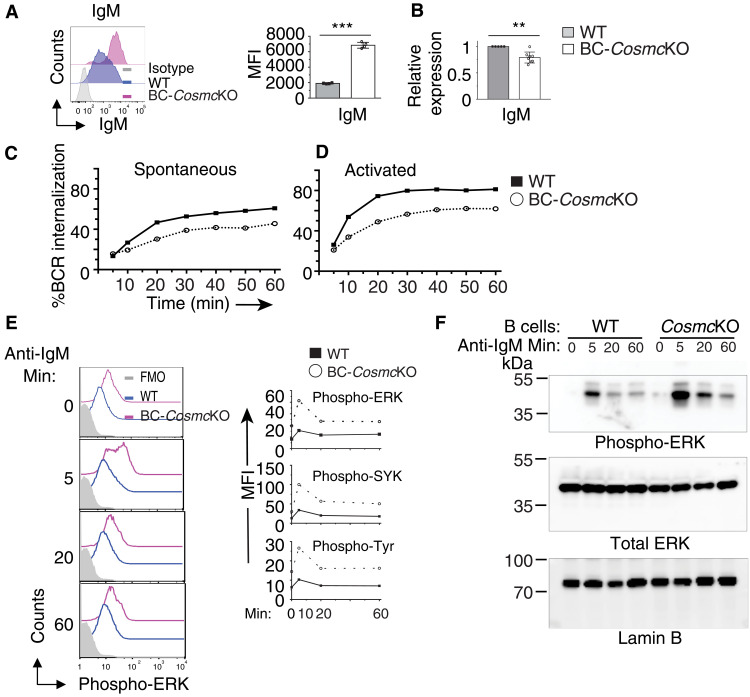
*Cosmc* deficiency leads to impaired BCR internalization and enhanced BCR signaling. (**A**) Representative histogram plot of IgM surface expression on B220^+^ B cells of both WT and BC-*Cosmc*KO mice. Right: MFI of IgM on B cells. Results are representative of three independent experiments with at least three animals in each group. (**B**) Transcript level of IgM in purified B cells of both WT and BC-*Cosmc*KO mice. *n* = 5 for WT and *n* = 8 for BC-*Cosmc*KO. (**C**) Spontaneous IgM internalization by WT and BC-*Cosmc*KO B cells incubated for indicated periods of time as measured by biotinylated Fab′ anti-IgM antibody. (**D**) Ligand-activated IgM internalization by WT and BC-*Cosmc*KO B cells incubated for indicated periods of time as measured by biotinylated F(Ab′)_2_ anti-IgM antibody. Experiments in (C) and (D) were repeated four times with similar results. Each symbol (black square, WT; open circle, BC-*Cosmc*KO) represents an individual mouse. Graphed as means ± 1 SEM. Young (2 to 4 months old) male mice were used in (A) to (F). Unpaired two-tailed Student’s *t* tests were performed to determine statistical significance, **P < 0.01 and ***P < 0.001. (**E**) Phospho-flow cytometry analysis of WT and BC-*Cosmc*KO B cells stimulated with anti-IgM antibody for indicated periods of time, assessing the intensity of phosphorylated SYK, ERK1/2, and total cellular proteins (pan-tyrosine). Right: MFI of indicated proteins. Experiments were repeated four times with similar results. (**F**) Phosphorylated ERK1/2 Western immunoblotting of WT and BC-*Cosmc*KO B cells stimulated with anti-IgM antibody (10 μg/ml) for indicated periods of time. Experiments were repeated two times with similar results.

Consistent with our previous finding (*23*), we observed that the expression level of IgM was elevated on naïve *Cosmc*-deficient B cells (Fig. 5A). Using quantitative polymerase chain reaction (PCR), we observed a reduced IgM mRNA transcript level in *Cosmc*-deficient B cells (Fig. 5B). One possible explanation of such divergence could be a defect in BCR internalization as a result of *Cosmc* deficiency. To investigate the effect of *Cosmc* deficiency on BCR internalization, we first examined the constitutive endocytosis of IgM. Both WT and *Cosmc*-deficient B cells were labeled with biotinylated Fab′ anti-IgM monoclonal antibody, which is unable to activate B cells but internalized with the BCR (*45*, *46*). The cell surface BCR level was then measured before and after incubation at 37°C by flow cytometry. The results indicate that BCR internalization is markedly impaired in *Cosmc*-deficient B cell, compared to WT (Fig. 5C). Next, we evaluated BCR internalization in response to ligand stimulation by incubating B cells with biotinylated F(Ab′)_2_ anti-IgM monoclonal antibody, which activates B cells by cross-linking surface BCR, in a T cell–independent manner (*46*, *47*). We observed that *Cosmc*-deficient B cells needed longer stimulation times to reach the similar levels of BCR internalization as in WT B cells (Fig. 5D). However, using the same stimulation time, the degree of BCR internalization in *Cosmc*-deficient B cells was consistently lower than that in WT B cells at all time points examined. Thus, *Cosmc* is required for both spontaneous and ligand-induced BCR internalization.

Upon ligand stimulation, some BCRs remain at the cell surface and cooperate with other co-receptors to initiate signal transduction, whereas the other BCRs are rapidly internalized (*48*). Thus, we reasoned that sustained surface BCR retention on *Cosmc*-deficient B cells could be associated with dysregulated BCR signals. To examine this possibility, we used a phospho-flow assay to analyze the phosphorylation kinetics of extracellular signal–regulated kinase 1/2 (ERK1/2), spleen tyrosine kinase (SYK), and total cellular proteins in both WT and *Cosmc*-null B cells after anti-IgM cross-linking stimulation. The basal phosphorylation levels of ERK1/2, SYK, and total cellular proteins were higher in *Cosmc*-deficient B cells, compared to WT B cells (Fig. 5E and fig. S6C). After anti-IgM activation, the up-regulation of phosphorylation levels of the aforementioned molecules continued and prolonged over time. We also observed enhanced activation kinetics of Erk1/2 in *Cosmc*-deficient B cells using phosphorylated protein Western immunoblotting (Fig. 5F). These results indicate that *Cosmc* deficiency causes impaired BCR internalization, which may lead to the observed increased surface IgM BCR levels, and subsequently enhanced BCR signaling and the resultant hyperresponsiveness of *Cosmc*-deficient B cells.

## DISCUSSION

By developing a mouse line in which *Cosmc* was specifically deleted in B cells, we found that the absence of core 1 O-glycans on B cells can spontaneously initiate the development of AID. In the mutated animals, we observed a high production of autoreactive antibodies, as well as a range of pathological alterations in multiple organs. We mechanistically show that the absence of core 1 O-glycans on B cells prolongs the surface retention of BCR that contributes to the enhanced BCR signaling. We also observed that *Cosmc*-deficient B cells display generalized hyperresponsiveness with regard to increased total serum IgM and IgG levels and spontaneous GCB cells. These findings represent a previously unappreciated critical role of glycosylation in the development of autoimmunity.

Glycosylation regulates multiple aspects of B cell biology, including B cell development, homing, and immunoglobulin effector function (*11*–*17*, *23*, *49*). Abnormally sialylated, galactosylated, and fucosylated N-glycans on glycoproteins such as immunoglobulins are often associated with multiple AIDs. However, how such aberrant glycosylation contributes to the pathogenesis of AID remains unanswered. Our study provides the first evidence that loss of core 1 O-glycans in B cells can trigger the development of AID (Fig. 1). We demonstrated that such glycosylation on B cells affects BCR signaling through regulating BCR surface retention (Fig. 5, C and D). Glycosylation on immune cells and molecules is known to be malleable to environmental exposure and developmental signals. Our study suggests that such factors may be able to skew immune cells, such as B cells, toward autoreactive pathway through altering O-glycosylation modification. In particular, it is important to note that reversible epigenetic hypermethylation of the promoter for *Cosmc* is known to arise in multiple disease conditions, including AID (*50*). Altered gene methylation is a known epigenetic mechanism involved in immune cell function in autoimmunity, including systemic lupus erythematosus (SLE) (*51*). In addition, the association of pathogen infections and AID pathogenesis has been suggested by epidemiological studies (*52*, *53*). However, the mechanism underlying such correlation remains elusive. It is noteworthy that pathogens can alter cellular glycosylation, as in sepsis, by introducing deleterious glycosidases, such as sialidases, which are also associated with many viral infections, e.g., influenza. Our study thus raises a plausible but rarely explored possibility that altered O-glycosylation on B cells could lead to unfavorable development of lymphocytes via the modification of their glycomes.

Our study also suggests that the absence of core 1 O-glycans on B cells affects the development of B cell–mediated AID through multiple mechanisms. Hyperresponsiveness of B cells is frequently associated with B cell–mediated autoimmunity (*2*, *37*). Mice defective in regulators of BCR signaling, such as CD22, FcγRIIb, and other molecules, generally demonstrate hyperactivation and/or hyperproliferation of B cells, enhanced BCR signaling, and AID-like pathology (*54*–*60*). In a related fashion, *Cosmc*-deficient B cells exhibit increased basal activation, as well as responsiveness to stimuli (Fig. 3), suggesting an inhibitory role of core 1 O-glycans on BCR signaling. We observed more IgM^+^ IgD^−^ immature B cells in the bone marrow in BC-*Cosmc*KO mice (*23*). Notably, B cell tolerance is generally believed to be established in immature B cells. The high percentage of *Cosmc*-deficient immature B cells therefore likely allows for B cells to be checked by various tolerance mechanisms, such as BCR editing or revision, and clonal deletion, to remove autoreactive B cells (*6*, *61*). However, the high amounts of autoreactive antibodies in BC-*Cosmc*KO mice clearly demonstrate that *Cosmc* deficiency leads to a breakdown of tolerance. It remains to be ascertained when and where the tolerance mechanisms are disrupted in *Cosmc*-deficient B cells. In addition, the much higher MHC II molecule levels on *Cosmc*-deficient B cells suggest that they might contribute to the autoimmunity development in additional ways, such as enhanced presentation of self-antigen to T cells. Furthermore, as CD9 is also a marker for B1 and plasma cells (*62*), the up-regulated expression of CD9 on *Cosmc*-deficient B cells suggests that loss of *Cosmc* may poise the B cells to differentiate toward those populations.

An important unaddressed issue in B cell biology is the functional relevance of rapid enhanced high PNA binding, which identifies core 1 O-glycans, when a subpopulation of antigen-experienced B cell enters GCB cell pathway. Among the surface molecular markers that have been defined and characterized in GCB cells, the PNA binding is especially noteworthy because it was the first marker used to identify GCB cells four decades ago, and it is still widely used (*43*, *44*, *63*). However, its functional relevance remains as “the still unsolved germinal center mystery” (*64*). PNA preferentially binds to nonsialylated core 1 O-glycans with a terminal galactose residue, e.g., T antigen, as evidenced by the ability of soluble galactose to block the binding. In line with this, we have performed glycan microarray experiments that demonstrate that the T antigen is preferentially recognized by PNA. As expected, B cells carrying a deletion of *Cosmc* exhibited substantially reduced PNA binding (Fig. 4A). We have also reported the presence of abnormal B cell subpopulations in the bone marrow and periphery of BC-*Cosmc*KO mice (*23*). Together, the results indicate a regulatory role of PNA receptors in B lymphocyte development.

To date, only a handful of surface makers—including PNA, Fas, CD38, and GL7—have been used to define GCB cells in both humans and mice (*65*–*68*). The latter three markers are directly or indirectly identified by characterizing the PNA^+^ GCB cells. Nonetheless, their roles in the development of GCB cells are rather mild. Our current studies on genetic deletion of the *Cosmc* show that the core 1 O-glycan is the main ligand of PNA on GCB cells (Fig. 4B). Moreover, loss of core 1 O-glycans on B cells appears to unleash the GCB cell response, and the percentage of GCB cell rises up to 10 times higher than that in WT mice (Fig. 4C), suggesting a negative role for core 1 O-glycans in the development of GCB cells. Furthermore, even the overall NP-specific IgG titers are similar in WT and BC-*Cosmc*KO mice, which suggests that immunoglobulin class switching was not affected in B cells’ loss of core 1 O-glycans. However, the titer of high-affinity (anti-NP_2_) antibody was lower in BC-*Cosmc*KO mice, suggesting a regulatory role of core 1 O-glycan in antibody affinity maturation process.

Our results indicate that BC-*Cosmc*KO mice represent a new animal model for AIDs based on several lines of evidence. First, a spectrum of AID clinical features spontaneously arises in BC-*Cosmc*KO mice, including weight loss, skin lesions, splenomegaly, hypergammaglobulinemia, and lymphadenopathy (*23*) (Figs. 1 and 2). We also found other disease manifestations such as ocular abnormalities and liver pathology, which are frequently found in human AIDs but not often seen in other animal models. Although the pathogenic mechanisms underlying these diseases remain to be investigated, it would be informative to test strategies to treat or alter the progression of the observed diseases, such as immunosuppression and administration of murine or human intravenous immunoglobulin. Moreover, the differential HEp-2 immunostaining patterns observed in BC-*Cosmc*KO mice indicated that *Cosmc* deficiency in B cells resulted in a diverse repertoire of self-reactive Tn^+^ B cell clones that have the capacity to readily differentiate and secrete destructive autoantibodies. Further exploration of factors that induce the differentiation of Tn^+^ B cells will help to understand the etiology of B cell–mediated AIDs. The causal relationship between elevated autoantibodies and the pathologies has not yet been established. An adoptive transfer experiment of serum or purified antibodies from BC-*Cosmc*KO mice into recipients should be considered next, although B cells might also contribute to the pathogenesis of AIDs in an antibody-independent manner. Second, many of the pathological phenotypes occurred in BC-*Cosmc*KO mice follow a female bias, mirroring the prevalence of most AIDs in females. Therefore, how gender-related factors, such as hormones, affect the initiation and progression of AID could be pursued using this mouse line.

In addition to our finding of compromised BCR signaling in B cells from BC-*Cosmc*KO mice, the molecular mechanisms by which O-glycans may regulate B cell activities remain to be studied. Previous studies show that the retention of BCR on cell surface is modulated by multiple factors (*46*, *69*, *70*). Uncoupling of CD19, one of the BCR co-receptors, resulted in altered BCR internalization via limiting its access to lipid rafts. Mutation of Igβ (CD79b) also changed the BCR internalization in a murine model. The retaining surface BCRs can be inductively phosphorylated and initiate signaling (*48*). Notably, many B cell surface molecules are predicted to be O-glycosylated, including CD19 and Igβ. It is possible that loss of O-glycans on these molecules may alter the interactions between BCR and endocytosis-related molecules and thereof contribute to the altered BCR surface retention, resulting in increased surface IgM levels, and the subsequent enhanced BCR signaling, as measured by increased phosphorylation of ERK. However, the O-glycosylated proteins on murine B cells are currently not defined and represent a substantial O-glycoproteomic challenge. It is yet to be elucidated regarding how the BCR downstream effectors are intertwined to determine the differentiation of *Cosmc*-deficient B cells. Nonetheless, *Cosmc* deficiency seems to lower the activation threshold in B cells, as manifested by their hyperresponsiveness to multiple stimuli (Fig. 3). On the other hand, we found evidence of the activation of immune effectors including increased inflammatory cytokine and potential activated complement pathways in the aged female BC-*Cosmc*KO mice (fig. S3). Such findings are reminiscent of those described in AID patients and animal models (*33*, *35*). Whether and/or how such factors contribute to the tissue damage and, if so, how to influence these factors to prevent injury will be intriguing to pursue further.

Overall, our results demonstrate that BC-*Cosmc*KO mice spontaneously develop AID-like pathologies. Thus, *Cosmc* and its cognate core 1 O-glycans on B cells act as an important checkpoint to prevent the development of pathogenic autoreactive B cells. Our study points to a model that *Cosmc* and core 1 O-glycans maintain B cell functional homeostasis by regulating BCR internalization. Upon loss of *Cosmc*, restricted internalization of the BCR and its consequent elevation on the cell surface are associated with enhanced BCR signaling, indicating that *Cosmc* negatively regulates BCR signaling. In line with this, the *Cosmc* mutant animals displayed a high production of autoreactive antibodies, as well as a range of pathological alterations in multiple organs. Our results are also consistent with recent studies implicating *Cosmc* in several autoimmune and inflammation-associated diseases, including inflammatory bowel diseases (*24*), IgAN (*25*), Tn syndrome (*26*), and Alzheimer’s disease (*27*). IgAN is particularly interesting as it has been well documented that Tn antigen expresses on the hinge region of IgA1, as well as enhanced production of autoantibodies to the altered glycoform and other autoantigens in patients with IgAN (*71*–*77*). In addition, a study suggests that in some IgAN patient cohorts, there is compromised expression of *Cosmc* (*25*). Thus, it is plausible that Tn^+^ antibody-secreting B cells exist in patients with IgAN and, perhaps, in patients with other autoimmune disorders.

While much remains to be explored in regard to the molecular and genetic basis underlying most AIDs, our results suggest the possibility that some type of dysglycosylation may induce B cell–mediated autoimmunity, and the potential for Tn^+^ B cells in this process should be considered. In terms of glycoprotein functions, it is now well documented that normal O-glycans are required in many systems for cell-cell interactions, normal glycoprotein stability, oligomeric complex formation, and cell signaling (*78*–*81*). The mouse model that we have presented here, in which autoimmunity spontaneously develops, suggests a potential relevance and mechanism of Tn antigen in the induction of AIDs. In summary, our results demonstrate an essential role of *Cosmc*, a regulator controlling O-glycosylation, in maintaining B cell tolerance and prevention of autoimmunity. These discoveries provide novel insights into the pathogenesis of AIDs and the potential to develop new diagnostic and therapeutic strategies for these diseases.

## MATERIALS AND METHODS

### Mice

*Cosmc*^f/f^ females were generated from our previous study (*19*) and bred with *Mb1*-Cre transgenic male mice (from M. Reth, Max Planck Institute of Immunobiology) to generate B cell–specific *Cosmc* knockout (BC-*Cosmc*KO) mice, and male BC-*Cosmc*KO mice were crossed with *Cosmc*^f/f^ females to generate female BC-*Cosmc*KO mice, which were cohoused with WT littermate controls under specific pathogen–free conditions (21.7° ± 0.6°C, 45 ± 10% humidity, and 12-hour light cycle of 6:00 a.m. to 6:00 p.m.) at Harvard Medical School in accordance with approved Institutional Animal Care and Use Committee protocols (Beth Israel Deaconess Medical Center, Harvard Medical School). Mice are on a C57BL/6 genetic background and age- and gender-matched throughout all experiments. Young male mice at 2 to 4 months old were used in functional study, and for characterization of spontaneous autoimmune phenotypes, old mice were used at 11 to 20 months old, unless stated otherwise. All mice were euthanized by carbon dioxide overdose in a euthanasia chamber. Mouse genotypes were determined by PCR with primers for *Mb1*-Cre [hCre dir, 5′-CCCTGTGGATGCCACCTC-3′ (forward); hCre, 5′-GTCCTGGCATCTGTCAGAG-3′ (reverse)] and *Cosmc^flox^* [5′-GCAACA CAAAGAAACCCTGGG-3′ (forward) and 5′-TCGTCTTTGTTAGGGGCTTGC-3′ (reverse)]. In addition, phenotyping of mice is also performed using anti-Tn and anti-CD19 antibodies by flow cytometry, where BC-*Cosmc*KO B cells are Tn positive and WT B cells are Tn negative.

### B cell isolation, antibodies, and flow cytometry

B cells were isolated from the spleen of WT and BC-*Cosmc*KO mice using a B cell isolation kit (Miltenyi Biotec, catalog no. 130-090-862) with a purity of >95%, as determined by CD19 positivity using flow cytometry. The antibodies were purchased from BD Biosciences, BioLegend, and eBioscience and listed as follows: CD19, B220, CD23, CD21, CD38, CD9, CD1d, Fas, GL7, phospho-ERK, phospho-SYK, phospho-tyrosine, IgM, IgD, CD86, CD80, and MHC II, conjugated to fluorophores as noted including FITC, PE, PE-Texas Red, PerCp, PE-Cy7, APC-Cy7, Brilliant Violet, Alexa Fluor 700, or Pacific Blue. Antibodies used in ELISA are from SouthernBiotech or Thermo Fisher Scientific. Anti-Tn antibody conjugated to Alexa Fluor 647 was prepared in the laboratory (*82*) according to the manufacturer’s protocol (Thermo Fisher Scientific, A20173). Biotinylated lectins were obtained from Vector Laboratories and incubated at a final concentration at 2 μg/ml. Single-cell suspensions prepared from spleen were stained with indicated antibodies at 1:100 dilution for 30 min and run on BD Biosciences FACSCalibur, LSRII, or CytoFLEX.

For TNP-Ficoll binding assay, mice were intravenously injected with TNP-Ficoll. Spleens were harvested after 30 min. Single-cell suspensions were prepared for biotin–anti-TNP (BD Biosciences, catalog no. 553978) staining, then streptavidin Alexa Fluor 488 (Life Technologies, S32354) staining, and flow cytometric analysis.

For in vitro cell culture, splenocytes prepared from both WT and BC-*Cosmc*KO mice were mixed and labeled with CellTrace Violet dye and stimulated with F(Ab′)_2_ fragment goat anti-mouse IgM (10 μg/ml; Jackson ImmunoResearch Laboratories, catalog no. 115-006-020) with or without the presence of anti-CD40 (1 μg/ml; eBioscience, catalog no. 16-0402-85) or ODN 1826 (1 μg/ml; synthesized by IDT) in complete RPMI 1680 media [10% fetal bovine serum, 5 mM Hepes, penicillin-streptomycin (50 U/ml), 2 mM l-glutamate, and 5 mM 2-mercaptoethanol]. After indicated periods of time, cells were pelleted and subjected to flow cytometric analysis.

For phospho-flow cytometry, splenocytes were activated by anti-IgM and permeabilized by dropwise adding cold methanol while vortexing and incubated on ice for 30 min. After extensive washing, the treated splenocytes were stained with anti–phospho-proteins and other surface markers and subjected to flow cytometric analysis. Data were analyzed with FlowJo software.

### Urinalysis

Urine was collected by holding the mouse over a collection container, and collected urine was subjected to a Chemstrip test (Roche, reference no. 11893467). A visual comparison of the results to the provided color scale was performed and scored as follows: (i) for presence of protein, below the limit of detection as 0 (negative), <30 μg/ml as 1, 30 to 100 μg/ml as 2, 100 to 500 μg/ml as 3, and more than 500 μg/ml as 4; (ii) for presence of glucose, below the limit of detection as 0 (normal), <50 μg/ml as 1, 50 to 100 μg/ml as 2, 100 to 250 μg/ml as 3, and 250 to 500 μg/ml as 4; (iii) for presence of nitrite, below the limit of detection as 0 (negative) and above the limit of detection as 1 (positive); (iv) for presence of leukocytes, below the limit of detection as 0 (negative), a “trace” finding as 1, a “+” finding as 2, and a “++” finding as 3; (v) for presence of blood/hemoglobin, below the limit of detection as 0 (negative), a trace finding as 1, an about 50 erythrocytes/μl finding as 2, and an about 250 erythrocytes/μl finding as 3. For bicinchoninic acid protein assay (Pierce, catalog no. 23225), urine was collected at two time points with a 6-hour interval. All samples were analyzed in duplicate, and the average of concentration measured from both time points was shown.

### Histopathology

Mouse liver tissues were collected and fixed in 10% formalin, and liver pathology was evaluated with hematoxylin and eosin (H&E) slides (processed by Beth Israel Deaconess Medical Center histology core facility) by two pathologists independently.

#### Kidney histopathology analysis

Kidneys were embedded in paraffin, sectioned onto slides, stained with H&E, examined microscopically, and scored as follows: (i) presence of crescents, fibrosis, or other morphological abnormalities, including hypertrophy and membranous thickening, in glomeruli (1 to 10%, grade 1; 10 to 25%, grade 2; 25 to 50%, grade 3; and >50%, grade 4); (ii) tubular cell changes (1 to 10%, grade 1; 10 to 25%, grade 2; 25 to 50%, grade 3; and >50%, grade 4); (iii) lymphocyte infiltration (1 to 10% of renal parenchyma, grade 1; 10 to 25%, grade 2; 25 to 50%, grade 3; and >50%, grade 4); and (iv) vasculitis (grade 1, small but definite perivascular infiltrates; grade 2, one to three foci of perivascular infiltrates without necrosis; grade 3, three to five foci of perivascular infiltrate, more extensive; and grade 4, more than five foci of perivascular infiltrates). The sum of the four histopathology scores was calculated. The glomerular index refers to the score of category (i) (*83*, *84*).

### Immunizations and ELISA

Eight-week-old WT and BC-*Cosmc*KO mice were immunized intraperitoneally with 100 μg of NP-KLH (Biosearch Technologies, N-5060-5) emulsified in complete Freund’s adjuvant, 10 μg of NP-Ficoll (Biosearch Technologies, F-1300-10), or NP-LPS (Biosearch Technologies, N-5065-1) in phosphate-buffered saline (PBS). Sera were collected from both immunized groups at indicated time points and stored at ≤−20°C.

For quantitative ELISA, sera from both WT and BC-*Cosmc*KO mice were collected, titrated, and added into 96-well plates (Corning) in duplicate that were precoated with polyvalent goat antibody against mouse immunoglobulins (IgM, IgG1, IgG2b, IgG2c, IgG3, and IgA from SouthernBiotech), next followed by horseradish peroxidase (HRP)–conjugated goat anti-mouse IgG (SouthernBiotech and Thermo Fisher Scientific), and then developed by trimethylboron (TMB) ELISA substrate (Abcam). Absorbance [optical density (OD)] value was determined at 450 nm with a Multiskan Spectrum spectrophotometer (Thermo Fisher Scientific). The concentration of mouse immunoglobulins was calculated from a standard curve constructed using mouse polyclonal immunoglobulins as listed: IgM, IgG1, IgG2b, IgG2c, IgG3, and IgA (SouthernBiotech).

Indirect ELISA was used to determine NP-specific immunoglobulins. Ninety-six–well plates were precoated with nitrophenol conjugated with bovine serum albumin (NP-BSA) or dsDNA or ssDNA overnight. For DNA-specific IgG, plates were first coated with protamine overnight before the coating with DNA. Plates were then washed and blocked with PBS/BSA (2%) for 90 min at room temperature (RT). Sera collected from immunized WT and BC-*Cosmc*KO mice were titrated in serial threefold dilution, added to the plates, and incubated at RT for another 90 min. After extensively washing, HRP-conjugated goat antibodies against mouse IgM, IgG, IgG1, IgG2b, IgG2c, or IgG3 were added to the plates for 90 min. Plates were washed thoroughly and developed by TMB ELISA substrate (Abcam). Absorbance (OD) value was measured at 450 nm with a Multiskan Spectrum spectrophotometer (Thermo Fisher Scientific). The NP-specific antibody titer is expressed as the reciprocal of the highest dilution as an absorbance value greater than twice that of the blank control in the same plate. The serum levels of the cytokines TNFα and IFNγ were measured by ELISA in accordance with the instructions of the manufacturer (R&D Quantikine ELISA kit, catalog nos. MTA00B and MIF00).

### HEp-2 staining and confocal microscopy

Antinuclear antibodies were examined by staining of Kallestad HEp-2 slides (Bio-Rad) with mouse serum (1:90 dilution), according to the manufacturer’s protocol, and followed by anti-mouse IgG conjugated to Alexa Fluor 488 and IgM conjugated to Alexa Fluor 568. Kidneys were harvested from both WT and BC-*Cosmc*KO mice and frozen in OCT at −80°C. The frozen tissues were sectioned at 6 μm thickness. The slides were then air-dried and fixed with cold 1:1 methanol/acetone fixative at −20°C for 10 min. After extensive washing with PBS containing 0.05% Tween 20, slides were blocked with 10% goat serum for 2 hours and then stained with anti-mouse IgG conjugated to Alexa Fluor 488 (Invitrogen, A10001) and IgM conjugated to Alexa Fluor 568 (Invitrogen, A21043) overnight. The slides were counterstained with Hoechst 33342 (Thermo Fisher Scientific, H3570) to visualize the nuclei, mounted with ProLong gold reagent (Invitrogen, P36930), and analyzed with a Zeiss LSM880 confocal microscope to acquire tile scanned images, which were then analyzed by ImageJ (Fiji).

### BCR internalization

Splenocytes were preequilibrated on ice in RPMI 1640 with 0.5% BSA and incubated with either biotinylated goat anti-mouse IgM F(ab′) (Jackson ImmunoResearch Laboratories, catalog no. 115-067-020) or F(Ab′)_2_ (10 μg/ml; Jackson ImmunoResearch Laboratories, catalog no. 115-006-020). After washing, the cells were then fixed in 0.5% paraformaldehyde in PBS (denoted as *T*_0_) or allowed for internalization at 37°C for the indicated periods of time (denoted as *T_n_*) before immediately fixed in 0.5% paraformaldehyde in PBS. The cells were then stained with streptavidin conjugated with Alexa Fluor 488, and the remaining BCRs on B cell surface were analyzed on a BD Biosciences FACSCalibur flow cytometer. The percentage of internalized BCR was calculated according to the following formula: 〔MFI IgM(*T*_0_) − MFI IgM (*T_n_*)/MFI IgM(*T*_0_) × 100.

### Quantitative real-time reverse transcription PCR

Total RNA was isolated from both WT and BC-*Cosmc*KO B cells using the RNeasy Mini Kit (QIAGEN, reference no. 74104) and dissolved in ribonuclease-free water. One microgram of total RNA was used to synthesize the first-strand complementary DNA using reverse transcriptase (SuperScript III; Invitrogen, reference no. 18080-044). Quantitative PCR was carried out using the SYBR Green PCR Master Mix (Bio-Rad) with CFX384 Touch Real-Time PCR Detection System (Bio-Rad), with primer IGHM 5′-GCTCAGCTATGCTACGCTGT-3′ (forward) and 5′-TGTTCTGGTAGTTCCAGGTGAA-3′ (reverse). Experimental Ct values were normalized to 36B4 or actin, and relative mRNA expression was calculated using the ddCt method.

### BN-APAGE analysis for immune complexes

Potential circulating IgG and IgM immunocomplexes from mice serum (BC-*Cosmc*KO and littermate control) were analyzed using BN-APAGE system (*32*). Briefly, BN-APAGE gels (0.8 to 12%), 2 mm thick, were prepared in a gel buffer containing 166 mM ε-aminocaproic acid and 50 mM bis-tris (pH 7.5) using 18 cm–by–8cm glass plates (Hoefer). Gels were poured at RT, superimposing decreasing gradient of agarose (Sigma-Aldrich, Type IX-A, ultralow gelling temperature) and an increasing gradient of acrylamide. Gels, with modified combs placed in them, were first kept at RT for acrylamide polymerization (~30 min) and then to 4°C to solidify agarose (1 hour). Comb from each gel was carefully removed and cleaned the wells by chilled 1× cathode buffer [50 mM tricine, 15 mM bis-tris, and 0.0015% G-250 (pH 7.0)]. Adding the cathode buffer on the interface of the gel and the comb and gently moving the comb against the glasses helps to remove comb smoothly.

Serum (2 μl; stored at −20°C and thawed on ice) was aliquoted and diluted to 398 μl of a dilution buffer [10 mM Hepes (pH 7.9), 1.5 mM MgCl_2_, 0.1 mM EDTA (pH 8.0), 0.1 mM EGTA (pH 8.0), and 10% glycerol]. The preparation (5 μl) of all the serum samples was mixed with sample buffer [0.5% Coomassie blue G-250 and 50 mM ε-aminocaproic acid in 10 mM bis-tris (pH 7.5) and 10% glycerol final concentration] just before loading, and electrophoresis was performed using 1× anode buffer [50 mM bis-tris HCl (pH 7.0)] and 1× cathode buffer for ~18 to 20 hours using relatively low voltage and low current (e.g., 16 to 20 V, ~2 mA, for four gels). Serum samples run on BN-APAGE gels were wet transferred at cold onto activated polyvinylidene difluoride (PVDF) membranes. After quickly destaining the membranes with 100% methanol, the membranes were washed with 1× TBST [50 mM tris-HCl (pH 7.4), 150 mM NaCl, and 0.05% Tween 20] and blocked with 1× TBST (5% nonfat milk) for 1 hour at RT. The preparation of the membranes was incubated for 1 hour at RT with HRP-labeled goat anti-moue IgM antibody (1:10,000) or goat anti-mouse IgG antibody (1:20,000) prepared in 1× TBST (5% nonfat milk). The membranes were washed five times for 5 min each and incubated with SuperSignal West Pico chemiluminescent substrate, and the signals were detected on the autoradiography films (HyBlot CL, Thomas Scientific). PVDF membranes were reblotted with enhanced chemiluminesence–anti-rabbit IgG and HRP F(Ab′)_2_ fragments (from donkey) (1:2000; 1× TBST, 1% nonfat milk) to locate 1.2-MDa native marker (Invitrogen, LC0725).

### Western immunoblotting

For phospho-protein Western immunoblotting, B cells purified from both WT and BC-*Cosmc*KO mice were treated with anti-IgM and pelleted and lysed in radioimmunoprecipitation assay buffer containing 150 mM NaCl, 50 mM tris-HCl (pH 7.6), 2 mM EDTA, 1% Triton X-100, and 0.1% SDS, supplemented with cOmplete Mini EDTA-free protease inhibitor cocktail (Roche) and PhosSTOP phosphatase inhibitors (Roche). Protein concentration was quantified by a DC protein concentration assay kit (Pierce) before subjecting to SDS-PAGE gel. Western blotting was performed with the following antibodies: pERK1/2 (Cell Signaling Technology, #4695T), lamin B (Cell Signaling Technology, #9622), and total ERK1/2 (Cell Signaling Technology, #9194).

For C3 complement Western blot analysis, sera were prepared from mice and stored at −20°C until use. Serum (1 μl) was loaded on SDS-PAGE and transferred to nitrocellulose membrane (Bio-Rad, catalog no. 1704158,) using Trans-Blot Turbo Transfer System (Bio-Rad). After blocking with 5% (w/v) nonfat milk (EMD Millipore, catalog no. C701K28) in TBST [50 mM tris-HCl (pH 7.4), 150 mM NaCl, and 0.05% Tween 20] for 1 hour at RT, the membranes were incubated with anti–complement factor 3 (C3) antibody (diluted at 1:2000; rabbit recombinant monoclonal IgG, catalog no. ab200999, Abcam) in TBST containing 1% nonfat milk for 1 hour at RT. After washing three times with TBST for 10 min, the membranes were incubated with HRP-labeled goat anti-rabbit IgG (KPL, catalog no. 374-1506) at 1:10,000 dilution in TBST containing 0.5% nonfat milk for 1 hour at RT. After washing three times with TBST for 10 min, the signals were analyzed on an Amersham Imager 600 (GE Healthcare Life Sciences) using SuperSignal West Pico chemiluminescent substrate (Thermo Fisher Scientific, catalog no. 34578).

### Statistics

For all applicable experiments, an unpaired two-tailed Student’s *t* test was performed for group comparisons using Prism software.
