# Supplementary material for: Cosmc deficiency causes spontaneous autoimmunity by breaking B cell tolerance
Source: Sci Adv. 2021 Oct 6;7(41):eabg9118. doi: 10.1126/sciadv.abg9118 (PMC8494437; doi:10.1126/sciadv.abg9118)
Supplement: Supplementary file 1 — Figs. S1 to S6 [file sciadv.abg9118_sm.pdf]

Science Advances
AAAS

Supplementary Materials for

Cosmc deficiency causes spontaneous autoimmunity by breaking B cell tolerance

Junwei Zeng*, Rajindra P. Aryal, Kathrin Stavenhagen, Chi Luo, Renyan Liu, Xiaohui Wang, Jiaxuan Chen, Hao Li, Yasuyuki Matsumoto, Yingchun Wang, Jianmei Wang, Tongzhong Ju, Richard D. Cummings*

*Corresponding author. Email: jzeng1@bidmc.harvard.edu (J.Z.); rcummin1@bidmc.harvard.edu (R.D.C.)

Published 6 October 2021, *Sci. Adv.* **7**, eabg9118 (2021)
DOI: 10.1126/sciadv.abg9118

This PDF file includes:

Figs. S1 to S6

Supplementary Figure Legends

Fig. S1 BC-*Cosmc*KO mice demonstrated autoimmune disease-like pathological features.

(**A**) Representative images of ocular manifestations of female (11-20 months old) and male mice (13-18 months old) from both BC-*Cosmc*KO and WT littermate controls shown. (**B**) Examples of ocular histology of male BC-*Cosmc*KO and WT mice by hematoxylin and eosin (H&E) staining. Scale bar represents 500 µm. (**C**) Summary of abnormal ocular manifestations in both male and female mice. (**D**) Wet weight of spleens of young female (left) at 2-4 months old, and aged male (right) at 13-18 months old. Each symbol (black square and open circle for WT and BC-*Cosmc*KO mice, respectively) represents an individual mouse, graphed as mean ± 1 standard error of the mean (SEM). Unpaired two-tailed Student's *t*-tests were performed to determine statistical significance with *** = $p<0.001$, ns = significant. (**E**) Steatosis of male mice from both BC-*Cosmc*KO and WT littermate controls at 13 to 18 months old. Liver sections were stained with H&E. Scale bar represents 100 µm. Representative images from both WT and BC-*Cosmc*KO mice were shown. Photo Credit: Junwei Zeng, BIDMC.

Fig. S2 Disrupted B cell tolerance in BC-*Cosmc*KO mice.

(**A**) Autoreactive antibodies of BC-*Cosmc*KO mice at 2 months old. Serum samples (1:90 dilution) from both BC-*Cosmc*KO and WT littermate controls were assayed by HEp-2 cell staining. Bound mouse IgM (red) detected by goat anti-mouse IgM conjugated with Alexa fluor-568, and bound IgG (green) detected by goat anti-mouse IgG conjugated with Alexa fluor-488. The WT-1 shown is the only one (1 out of 17) demonstrating minimal HEp-2 reactivity. Images were acquired at 63x magnification. Scale bar represents 20 µm. (**B**) Summary of assayed samples from BC-*Cosmc*KO mice. (**C**) Immunoglobulin deposition in kidneys of female BC-*Cosmc*KO mice. Kidney images acquired at 20x magnification. Representative images from both WT (n=5) and BC-*Cosmc*KO male mice (n=6) shown. Scale bar represents 50 µm. (**D**, **E**) Urine was collected from aged BC-*Cosmc*KO and WT mice of both genders for analysis by Chemstrips (**D**), and Bicinchoninic Acid (BCA) protein assay (**E**). WT (n=7) and BC-*Cosmc*KO mice (n=9) for male, WT (n=7) and BC-*Cosmc*KO mice (n=7) for female in **D**. WT (n=4) and BC-*Cosmc*KO mice (n=8) for male, WT (n=6) and BC-*Cosmc*KO mice (n=7) for female in **E**. The female urinary protein data (by BCA) are representative of two independent experiments with similar results, at least 6 animals in each group. (**F**, **G**) Histopathology scores of kidneys of both aged mice: WT (n=6) and BC-*Cosmc*KO mice (n=9) for male in **F**, and WT (n=8) and BC-*Cosmc*KO mice (n=12) for female in **G**. Representative images of H&E staining of kidneys of aged female shown and boxed area shown at x40 magnification in **G** (right). Scale

bars represent 100 μm (left) and 20 μm (right) respectively. The glomerular index is the score of the category (a), detailed in the Methods. (**H**) Blue native agarose polyacrylamide gel electrophoresis (BN-APAGE) analysis for IgM and IgG of both aged WT and BC-*Cosmc*KO mice sera. Four and three biological replicates of female and male respectively are shown. For **D-G,** each symbol (black square and open circle for WT and BC-*Cosmc*KO mice, respectively) represents an individual mouse, graphed as mean ± 1 standard error of the mean (SEM). Serum or tissues were collected from age-matched aged (11-20 months old) and young (2-4 months old) mice of indicated gender. Unpaired two-tailed Students *t*-tests were performed to determine statistical significance with ns stands for non-significant, * = p<0.05, ** = p<0.01.

Fig. S3 Immune effectors in BC-*Cosmc*KO mice.

Blood samples were collected from indicated groups for **A-D**. Determination of TNFα (**A**) and IFNγ (**B**) levels in sera were conducted by ELISA, according to manufacturer's instructions. Dashed lines represent the minimal detection dose, 1.88 pg/ml for TNFα, and 2 pg/ml for IFNγ. Detection of full length C3 complement and its breakdown fragments of young (**C**) and aged (**D**) mice was analyzed by Western blot. Asterisks in **D** indicates presence of aberrant complement C3 and breakdown subunits in the aged female BC-CosmcKO mice. Each symbol (black square and open circle for WT and BC-*Cosmc*KO mice, respectively) represents an individual mouse, graphed as mean ± 1 standard error of the mean (SEM). Sera were collected from age-matched aged (11-20 months old) and young (2-4 months old) mice of indicated gender. ns = not significant. Unpaired two-tailed Student's *t*-tests were performed to determine statistical significance with * = p<0.05, ** = p<0.01.

Fig. S4 Enhanced basal activation of *Cosmc*-deficient B cells.

(**A**) Expression levels of CD9 and CD1d on follicular B cells ($B220^{+}$ or $CD19^{+}$ $CD23^{+}$ $CD21^{lo}$ FOB, upper row) and marginal zone B cells ($B220^{+}$ or $CD19^{+}$ $CD23^{lo}$ $CD21^{hi}$ MZB, bottom row) by flow cytometry. Expression levels of CD86, CD80, and MHC II on (**B**) FOB cells and (**C**) MZ B cells. Splenocytes are prepared from both BC-*Cosmc*KO and WT littermate controls. The results are representative of three independent experiments with at least 3 animals in each group. Each symbol (black square and open circle for WT and BC-*Cosmc*KO mice, respectively) represents an individual mouse, graphed as mean ± 1 standard error of the mean (SEM). Cells were prepared from young (2-4 months old) male mice. Unpaired two-tailed Student's *t*-tests were performed to determine statistical significance with * = p<0.05, ** = p<0.01, *** = p<0.001.

Fig. S5 *Cosmc* deficiency reduces PNA binding on germinal center B cells.

Binding analysis of biotinylated PNA, MAL-II, SNA, and Con A, followed by streptavidin-Alexa fluor 488, on neuraminidase or PBS treated WT and BC-*Cosmc*KO splenic B cell subsets (**A**): follicular B cells ($B220^{+}$ or $CD19^{+}$ $CD23^{+}$ $CD21^{lo}$ FOB, top two rows) marginal zone B cells ($B220^{+}$ or $CD19^{+}$ $CD23^{lo}$ $CD21^{hi}$ MZB, bottom two rows). (**B**) Representative histogram plots of PNA binding of splenic germinal center B cells ($B220^{+}$ or $CD19^{+}$ $GL7^{+}$ Fas^{+}) and non-germinal center B cells ($B220^{+}$ or $CD19^{+}$ $GL7^{-}$ Fas^{-}). Right, mean fluorescence intensity (MFI) of PNA binding on indicated B cell population is shown for each WT and BC-*Cosmc*KO mice. (**C**) Representative FACS plots of the germinal center B cell population in MLN simultaneously stained with CD19, GL7, Fas, and CD38, and analyzed by two gating strategies ($CD19^{+}GL7^{+}$ Fas^{+} left, and $CD19^{+}CD38^{lo}$ Fas^{+} middle). Right, percentage of GC B cells of total B cells shown for WT and BC-*Cosmc*KO mice of indicated tissue. Each symbol (black square and open circle for

WT and BC-*Cosmc*KO, respectively) represents an individual mouse, graphed as mean ± 1 standard error of the mean (SEM). Cells were prepared from young (2-4 months old) male mice in **A** and **B,** and from young male and female mice in **C**. Unpaired two-tailed Student's *t*-tests were performed to determine statistical significance with ** = $p<0.01$, *** = $p<0.001$. ns = not significant.

Fig. S6 Antigen specific Immune responses in BC-*Cosmc*KO mice.

NP-specific immunoglobulin responses to immunization with (**A**) NP-LPS, (**B**) NP-Ficoll. (**A**) Both WT (n=9) and BC-*Cosmc*KO mice (n=11) were intraperitoneally immunized with NP-LPS. (**B**) Both WT (n=9) and BC-*Cosmc*KO mice (n=9) were intraperitoneally immunized with NP-Ficoll. ELISA determined the titers of NP-specific IgM (**A** and **B**) of sera collected from immunized mice at indicated time points. Data were from two experiments with similar results. (**C**) Phospho-flow cytometry analysis of WT and BC-*Cosmc*KO B cells stimulated with anti-IgM antibody for indicated periods of time. Representative histogram plots of phosphorylated SYK and total cellular proteins (pan-tyrosine) shown. Each symbol (black square and open circle for WT and BC-*Cosmc*KO, respectively) represents an individual mouse, graphed as mean ± 1 standard error of the mean (SEM). Young (2-4 months old) male mice were used in **A-C**. Unpaired two-tailed Student's *t*-tests were performed to determine statistical significance with ** = $p<0.01$.

Supplementary Figures

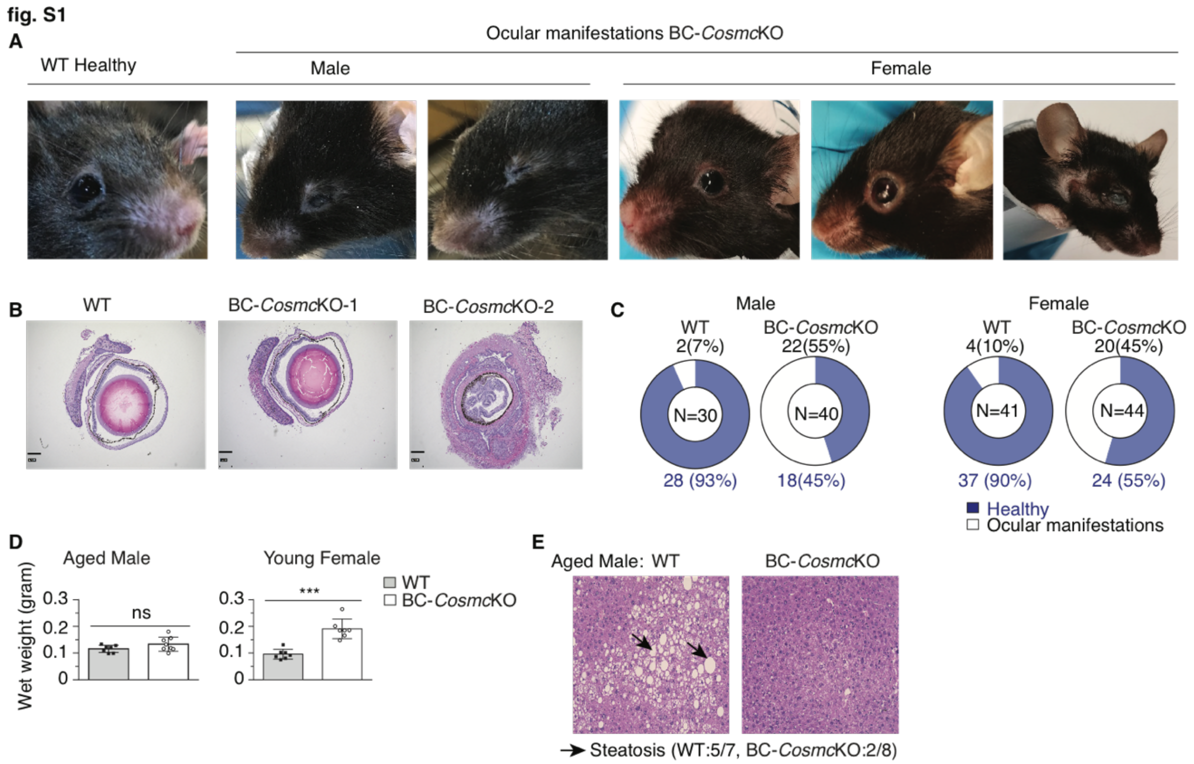

fig. S2

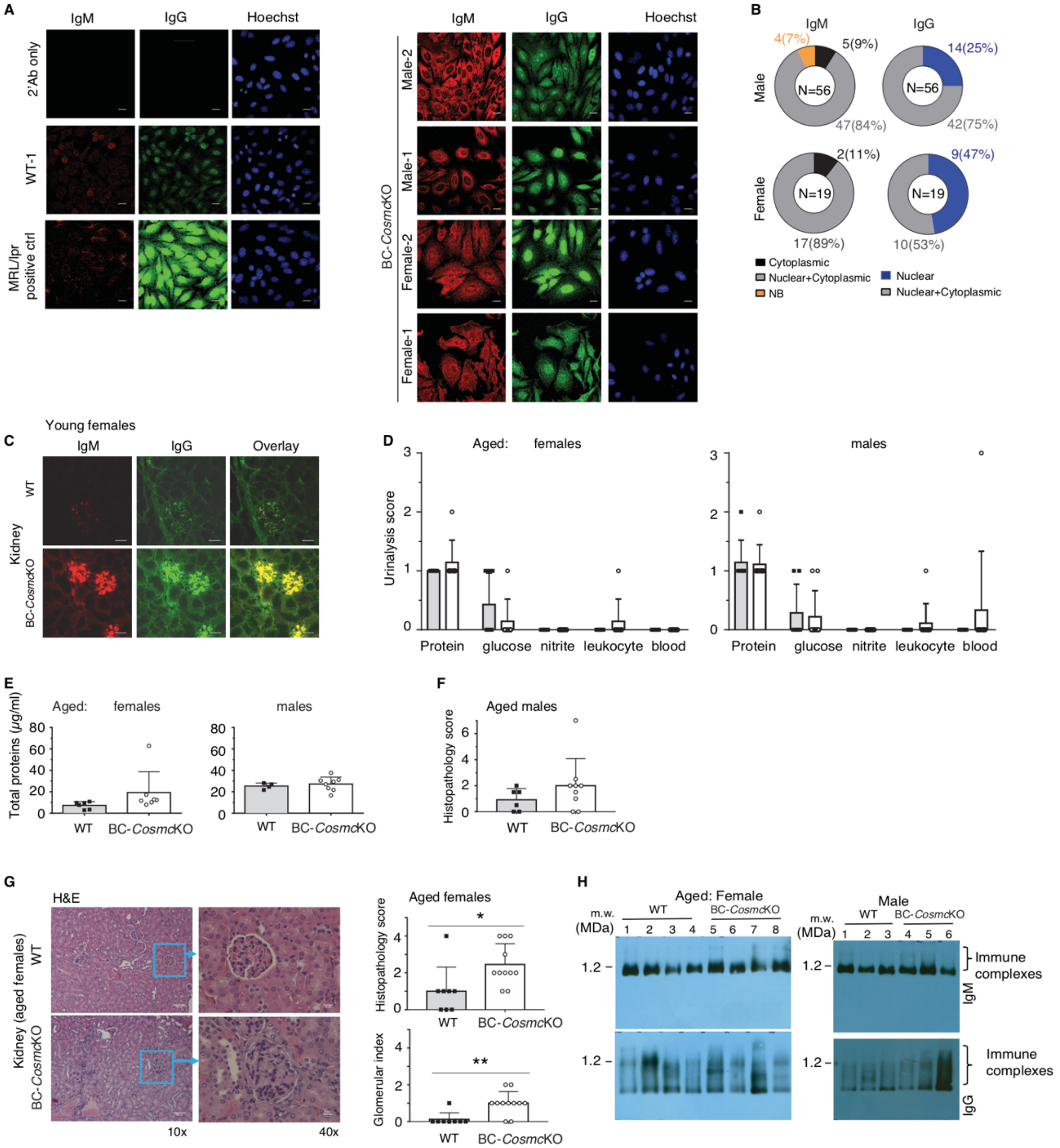
A
IgM
IgG
Hoechst
2'Ab only
WT-1
MRL/lpr positive ctrl
BC-CosmcKO
Male-2
Male-1
Female-2
Female-1
B
IgM
IgG
Male
Female
N=56
N=19
4(7%)
5(9%)
47(84%)
14(25%)
42(75%)
2(11%)
17(89%)
9(47%)
10(53%)
Cytoplasmic
Nuclear+Cytoplasmic
NB
Nuclear
Nuclear+Cytoplasmic
C
Young females
IgM
IgG
Overlay
Kidney
WT
BC-CosmcKO
D
Aged: females
males
Urinalysis score
Protein
glucose
nitrite
leukocyte
blood
E
Aged: females
males
Total proteins (μg/ml)
WT
BC-CosmcKO
F
Aged males
Histopathology score
WT
BC-CosmcKO
G
H&E
Kidney (aged females)
WT
BC-CosmcKO
10x
40x
Aged females
Histopathology score
Glomerular index
WT
BC-CosmcKO
H
Aged: Female
Male
m.w. (MDa)
WT
BC-CosmcKO
1.2
Immune complexes
IgM
IgG

fig. S3

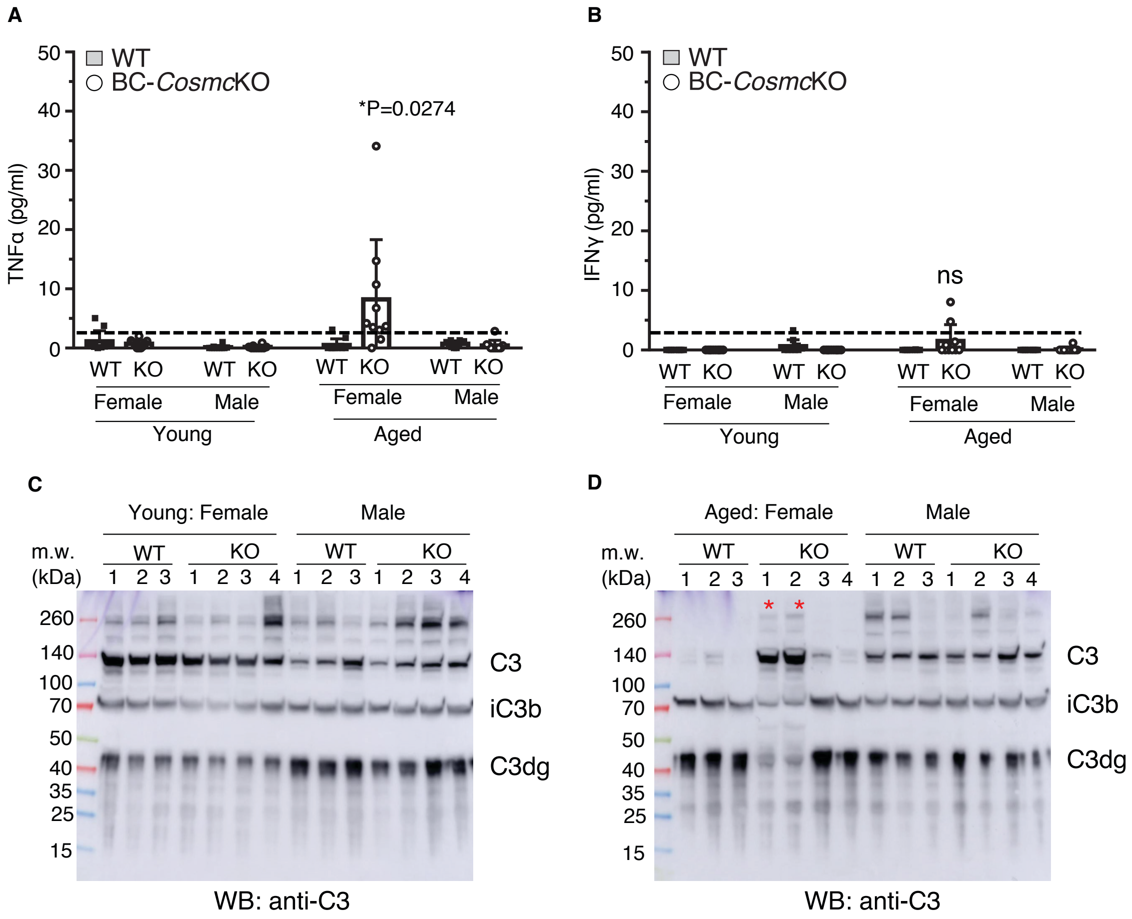
A
WT
BC-*Cosmc*KO
TNFα (pg/ml)
*P=0.0274
WT KO WT KO WT KO WT KO
Female Male Female Male
Young Aged
B
IFNγ (pg/ml)
ns
C
Young: Female
Male
m.w. (kDa)
260
140
100
70
50
40
35
25
15
C3
iC3b
C3dg
WB: anti-C3
D
Aged: Female
Male
WB: anti-C3

fig. S4

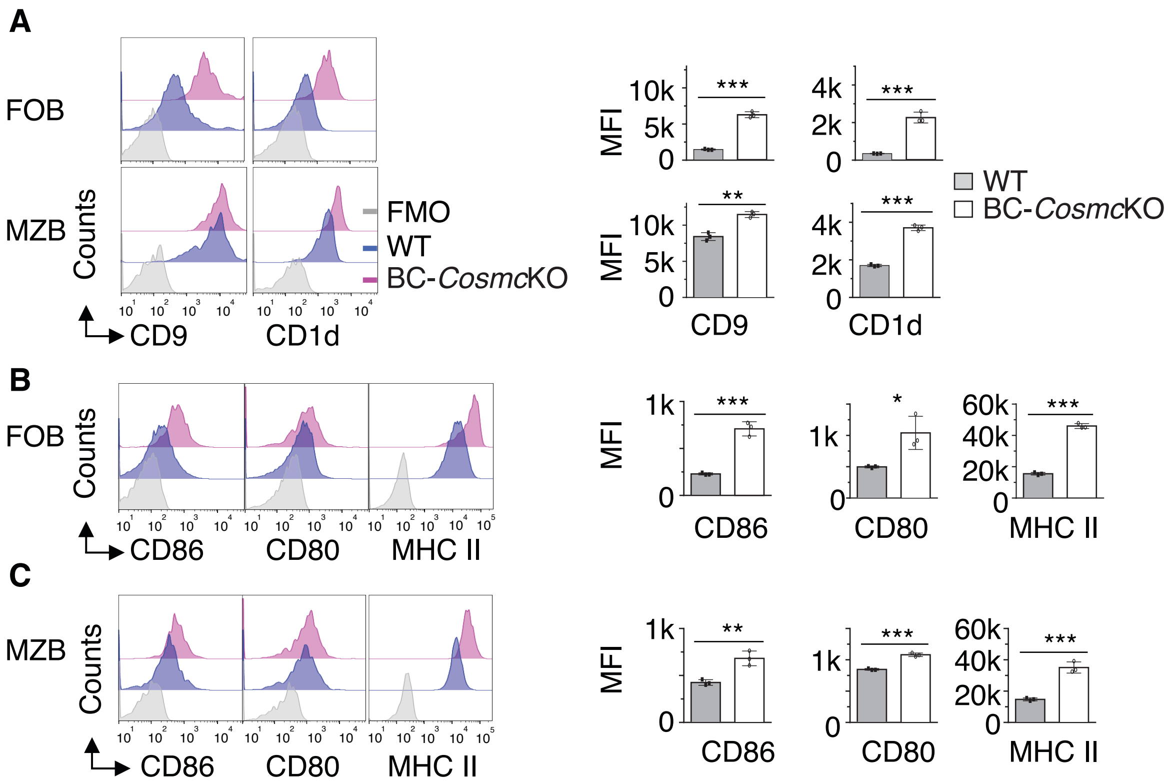
A
FOB
MZB
Counts
FMO
WT
BC-*Cosmc*KO
CD9
CD1d
MFI
10k
5k
0
4k
2k
***
**
WT
BC-*Cosmc*KO
B
FOB
Counts
CD86
CD80
MHC II
1k
60k
40k
20k
*
C
MZB
Counts
CD86
CD80
MHC II

fig. S5

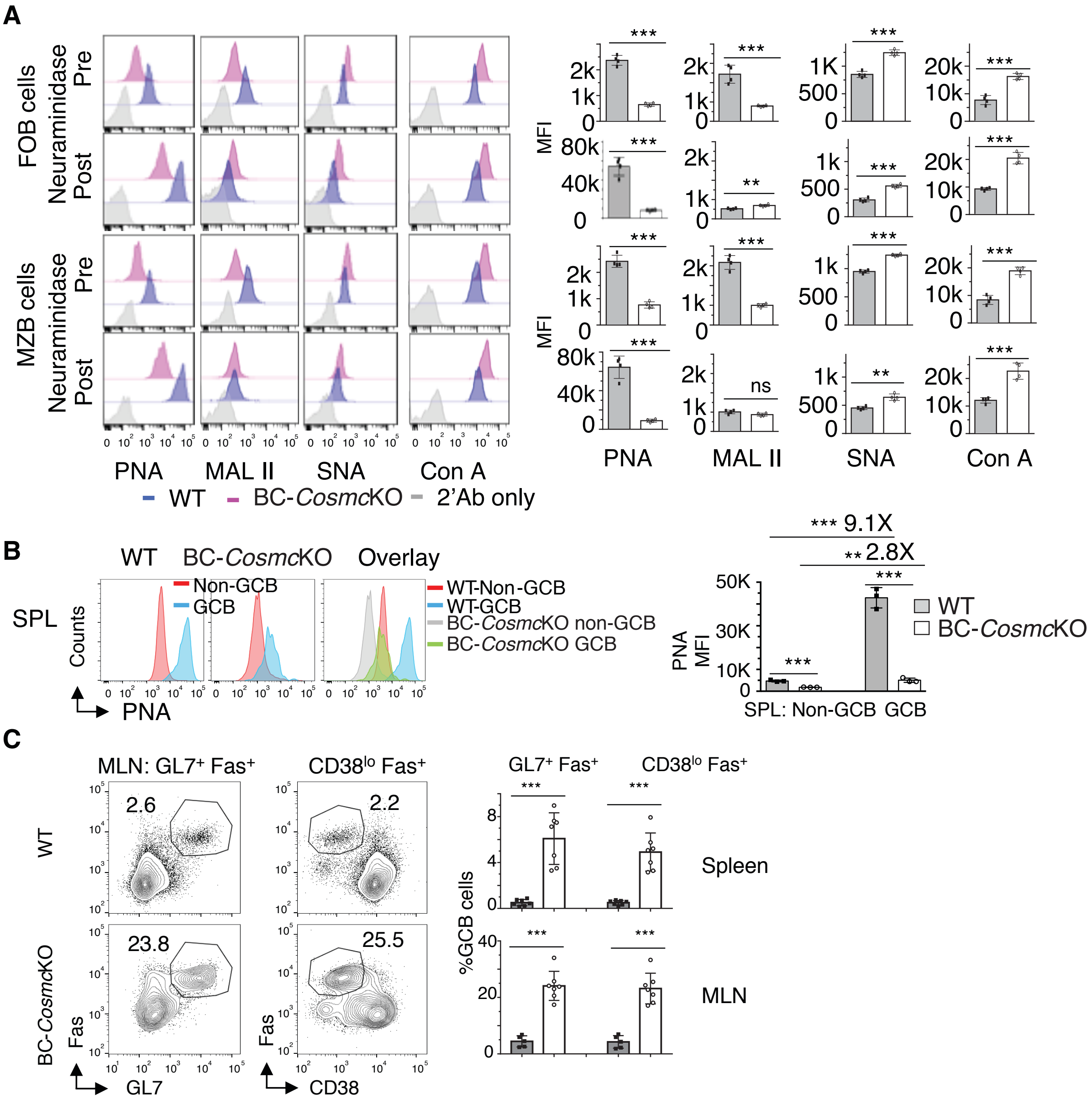
A
FOB cells
MZB cells
Neuraminidase Pre
Neuraminidase Post
MFI
PNA
MAL II
SNA
Con A
WT
BC-CosmcKO
2'Ab only
***
**
ns
B
WT
BC-CosmcKO
Overlay
Non-GCB
GCB
WT-Non-GCB
WT-GCB
BC-CosmcKO non-GCB
BC-CosmcKO GCB
SPL
Counts
PNA
*** 9.1X
** 2.8X
PNA MFI
SPL: Non-GCB GCB
C
MLN: GL7+ Fas+
CD38lo Fas+
2.6
2.2
23.8
25.5
Fas
GL7
CD38
GL7+ Fas+
CD38lo Fas+
%GCB cells
Spleen
MLN

fig. S6

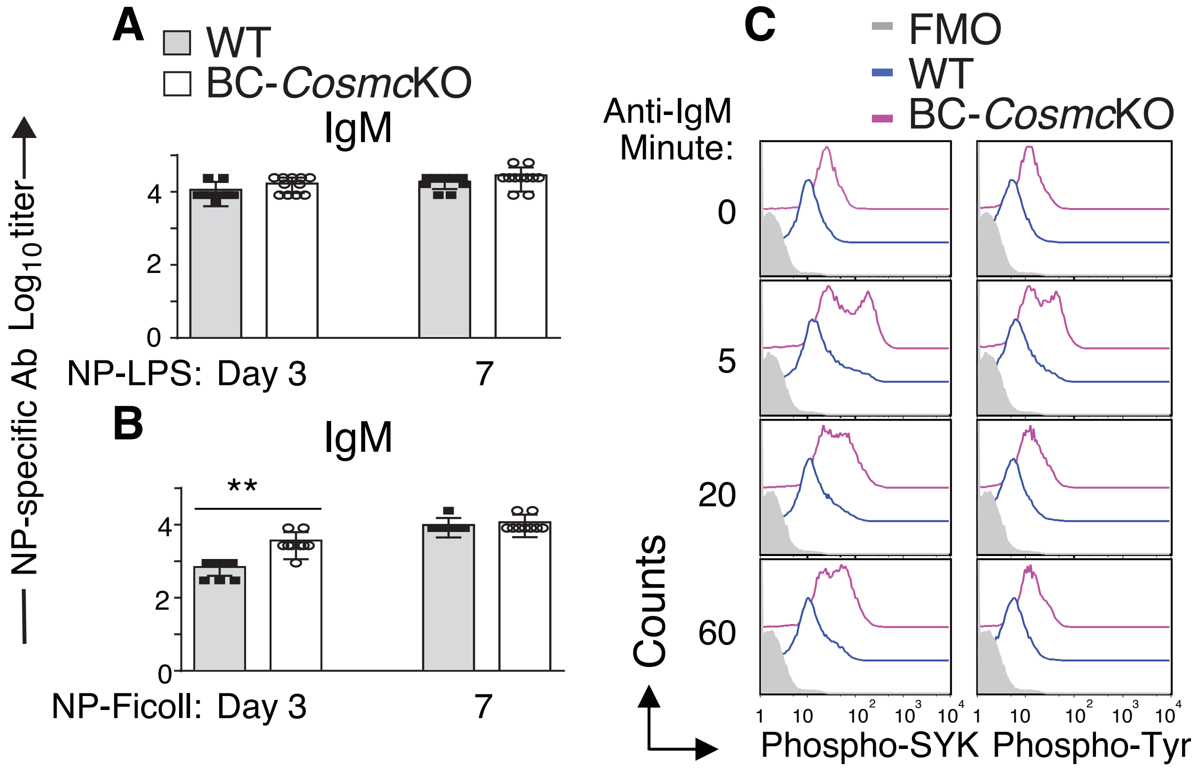

A
WT
BC-*Cosmc*KO
IgM
4
2
0
NP-LPS: Day 3
7
NP-specific Ab Log10 titer
B
IgM
**
4
2
0
NP-Ficoll: Day 3
7
C
FMO
WT
BC-*Cosmc*KO
Anti-IgM
Minute:
0
5
20
60
Counts
1 10 10^2 10^3 10^4
Phospho-SYK
Phospho-Tyr
